# Cultivation strategies for prokaryotes from extreme environments

**DOI:** 10.1002/imt2.123

**Published:** 2023-06-12

**Authors:** Zi‐Wen Yang, Zheng‐Han Lian, Lan Liu, Bao‐Zhu Fang, Wen‐Jun Li, Jian‐Yu Jiao

**Affiliations:** ^1^ State Key Laboratory of Biocontrol, Guangdong Provincial Key Laboratory of Plant Resources and Southern Marine Science and Engineering Guangdong Laboratory (Zhuhai), School of Life Sciences Sun Yat‐Sen University Guangzhou China; ^2^ State Key Laboratory of Desert and Oasis Ecology, Key Laboratory of Ecological Safety and Sustainable Development in Arid Lands, Xinjiang Institute of Ecology and Geography Chinese Academy of Sciences Urumqi China

**Keywords:** cultivation strategy, extreme environment, extremophiles, multi‐omics, pure culture

## Abstract

The great majority of microorganisms are as‐yet‐uncultivated, mostly found in extreme environments. High‐throughput sequencing provides data‐rich genomes from single‐cell and metagenomic techniques, which has enabled researchers to obtain a glimpse of the unexpected genetic diversity of “microbial dark matter.” However, cultivating microorganisms from extreme environments remains essential for dissecting and utilizing the functions of extremophiles. Here, we provide a straightforward protocol for efficiently isolating prokaryotic microorganisms from different extreme habitats (thermal, xeric, saline, alkaline, acidic, and cryogenic environments), which was established through previous successful work and our long‐term experience in extremophile resource mining. We propose common processes for extremophile isolation at first and then summarize multiple cultivation strategies for recovering prokaryotic microorganisms from extreme environments and meanwhile provide specific isolation tips that are always overlooked but important. Furthermore, we propose the use of multi‐omics‐guided microbial cultivation approaches for culturing these as‐yet‐uncultivated microorganisms and two examples are provided to introduce how these approaches work. In summary, the protocol allows researchers to significantly improve the isolation efficiency of pure cultures and novel taxa, which therefore paves the way for the protection and utilization of microbial resources from extreme environments.

## Highlights

- A straightforward protocol for efficiently isolating prokaryotic microorganisms from different extreme habitats is proposed.
- A bioinformatic tool for easily analyzing 16S rRNA gene sequences with the EzBioCloud database is developed.
- Multi‐omics‐guided microbial cultivation approaches are useful for culturing as‐yet‐uncultivated microorganisms.

## Graphical abstract

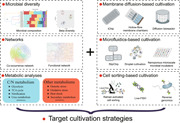

## INTRODUCTION

Extreme environments, including hot spring, hydrothermal vent, glacier, permafrost, desert, soda lake, saltern, and acid mine drainage sites, are significant geological forms on Earth [1, 2], which harbor unexpectedly diverse extremophiles from all three domains of life [3]. Because of the distinctive and harsh conditions, microorganisms living in these environments have typically evolved different strategies to cope with the extreme environmental stresses which define the boundary of life [4, 5, 6, 7]. These extremophiles can not only provide unique products (such as Taq polymerase [8]) but are also ideal targets for the study of microbial ecology, evolution, and environmental adaptation [9, 10, 11]. More than that, extremophiles are also regarded as optimal models to research the origin and evolution of life and even the potential for extraterrestrial life [12, 13, 14, 15].

In recent decades, with the development of sequencing technologies, multi‐omics studies have enabled researchers to obtain genetic information and glimpse the microbiome without microbial cultivation, which has greatly improved our understanding of microorganisms in extreme environments and changed the structure of the “tree of life” [16, 17, 18, 19, 20]. Culture‐independent surveys of naturally occurring microbial populations have revealed intriguing associations between the microbial communities and extreme environments, but these observed associations (including metabolic functions) are only the potential predicted by genomic information rather than direct evidence [3, 21, 22, 23]. Culture‐dependent methods, by contrast, not only can enable the recovery of complete reference genomes and the development of genetic manipulation systems of microorganisms but are also the means to comprehensively examine the physiology and enzymology of microorganisms [24, 25, 26]. Therefore, cultivating microorganisms from extreme environments is necessary to investigate the diversity, function, and evolution of extremophiles. For this reason, microbial cultivation is currently experiencing a pronounced revival [27, 28, 29, 30]. In recent years, some exciting cultivation results have greatly expanded our knowledge of life in extreme environments and even the origin of the Eukaryotes [31, 32, 33]. More than that, according to a number of case studies, culture‐dependent methods can also investigate rare microorganisms in microbial communities or those with difficult to extract DNA which can be difficult to detect by culture‐independent techniques [34, 35, 36, 37]. Therefore, culturing microorganisms from extreme environments is not only important but also necessary. To promote the isolation of extremophile microorganisms, in this article, we want to share our experiences and strategies with researchers working on isolating prokaryotic microorganisms from extreme environments.

## COMMON PROCESSES FOR EXTREMOPHILE ISOLATION

**Figure 1:**
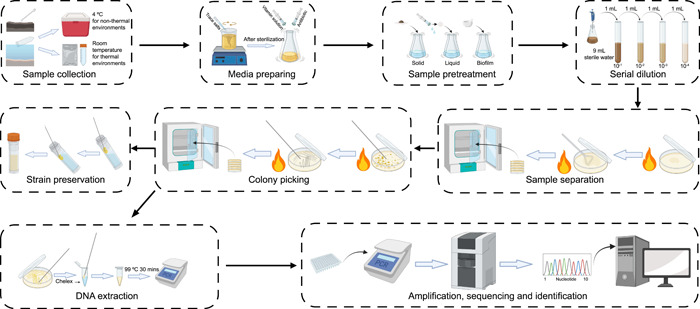
Nine steps common to the process for isolating microorganisms from extreme environments.

The common processes for the isolation of extremophiles can be divided into four parts and nine steps (Figure 1). Although many researchers already have some experience in isolating strains from extreme environments, there are still many aspects that are too often ignored that can influence the effectiveness of the isolation approach.

### Sample collection from extreme environments

Materials and devices: sterile scalpels and spades, sterile falcon tubes and Ziploc bags, Hungate‐type anaerobe tubes, ice bags, and refrigerator.

#### Sample collection

Different kinds of samples from normal temperature niches, such as salt lake, marine sediment, desert sand, acidic mine drainage (AMD), and so forth, are collected using sterile scalpels or spades and transferred immediately into falcon tubes or Ziploc bags. These kinds of samples are usually stored at a low temperature (4°C) for later isolation and analysis of physicochemical characteristics. The samples collected from hot springs are normally kept at room temperature, as this is useful for maintaining the activity of strains and better for subsequent isolation. For the anaerobic isolation, samples need to be kept in sterile Hungate‐type anaerobe tubes with a reducing agent such as cysteine or sodium sulfide. Resazurine, which is a redox dye, needs to be added to confirm that the sample has been reduced. The samples for DNA extraction are typically mixed and collected in sterilized falcon tubes and immediately stored in liquid nitrogen, then transported back to the laboratory and kept at −80°C before DNA extraction. *Note*: The microbial community in the samples preserved at 4°C will change over time. Therefore, according to the previous test [38] and the experience of former isolation work, we recommend the samples preserved at 4°C better to carry out the isolation work within 20 days.

### Media preparation and sample preprocessing

Materials and devices: Petri dish, spirit lamp, bacterial cell spreader, pipettes and tips, 15 mL centrifuge tubes, 100 mL conical flasks, glass beads, 0.22 μm filters, 10 mL injector, mortar and pestle, inorganic salts, multivitamins, nystatin and dimethylsulfoxide (DMSO), agar, precision balance, autoclave, orbital shaker, constant temperature incubator, and clean bench.

#### Media preparing

Choosing appropriate media is crucial in microbial isolation experiments. The recommended media for each extreme environment are listed in Table 1. Besides the media components themselves, trace salts and multivitamin solutions are also recommended to be added into different isolation media [39]. The recommended formula of trace salts is: MgSO_4_·7H_2_O 3 g/L, MnSO_4_·2H_2_O 0.5 g/L, NaCl 1 g/L, FeSO_4_·7H_2_O 0.1 g/L, CoCl_2_ 0.1 g/L, CaCl_2_·2H_2_O 0.1 g/L, ZnCl_2_ 0.1 g/L, CuCl_2_·2H_2_O 0.02 g/L, AlK(SO_4_)_2_ 0.01 g/L, H₃BO₃ 0.01 g/L, Na_2_MoO_4_·2H_2_O 0.1 g/L, NiCl₂·6H₂O 0.03 g/L, and Na_2_WO_4_·2H_2_O 0.03 g/L. Trace salts (10 mL) should be added per liter of medium. The recommended formula of multivitamin solution is: biotin 5 mg/L, folic acid 5 mg/L, pyridoxine hydrochloride 25 mg/L, riboflavin 12.5 mg/L, thiamine 12.5 mg/L, nicotinic acid 12.5 mg/L, pantothenic acid 12.5 mg/L, cobalamin 5 mg/L, *p*‐aminobenzoic acid 12.5 mg/L, and thioctic acid 12.5 mg/L. Multivitamin solution (2 mL) should be added per liter of medium. In addition, if there is no need to isolate fungi or algae, nystatin (DMSO solution, working concentration 0.05 mg/mL) can be added to different isolation media to inhibit the growth of fast‐growing eukaryotic microorganisms. The multivitamin solution and nystatin are both required to be filtered through 0.22‐μm filter membranes and added after sterilization of the media. When pouring plates, the height of medium is best exceeding 1/2 of the Petri dish and should be air‐dried overnight to remove surface moisture. *Note*: Agar is not suitable when the pH value of the media is too high/too low or the culture temperature is too high. These factors will destroy the hydrogen bond of the ager that makes it hard to solidify. Instead, gelrite has the ability of solidification at these situations and sometimes can get better isolation result [40]. When the medium contains phosphate, it is better to autoclave separately the phosphate and medium with agar, as this can greatly improve the cultivability of microorganisms by decreasing oxidative stress from hydrogen peroxide [41, 42, 43]. Preliminary experiments are sometimes necessary to establish suitable/optimum isolation media, given the highly varied sources from which samples are obtained. The former study and our experiences have shown that a medium which contains the same physical and chemical composition compared with the in situ environment of samples may not be the suitable/optimum condition for isolation, implying that many microorganisms may not be active in the original extreme environment or that additional signals are needed to activate growth [44].

**Table 1:** Recommended media for recovering strains from different extreme environments.

Medium	Composition (g/L)^a^	Applicable environment	Reference
R2A agar	Yeast extract 0.5, tryptone 0.5, casamino acids 0.5, glucose 0.5, soluble starch 0.5, K_2_HPO_4_ 0.3, MgSO_4_·7H_2_O 0.05, sodium pyruvate 0.3, agar 15.0, pH 7.5.	Thermal environment, xeric environment, saline and alkaline environment, cryogenic environment	Reasoner and Geldreich [45]
MM agar	Glucose 0.5, yeast extract 0.5, K_2_HPO_4_ 1.0, MgSO_4_·7H_2_O 0.5, NaCl, 0.5, agar 15.0, pH 7.5.	Xeric environment	Hozzein et al. [46]
Actinobacterial isolation agar	Sodium propionate 4.0, sodium caseinate 2.0, K_2_HPO_4_ 0.5, l‐asparagine 0.1, MgSO_4_·7H_2_O 0.1, FeSO_4_·7H_2_O 0.001, agar 15.0, pH 8.1.	Xeric environment	Li et al. [37]
T5 agar	Glucose 1.0, lotus root starch 1.0, yeast extract 2.0, tryptone 0.5, CaCO_3_ 0.5, agar 15.0, pH 7.5.	Thermal environment	Narsing Rao et al. [47]
YPXS agar	Yeast extract 5.0, peptone 1.0, oat spelt xylan 5.0, sulfur 10.0, PIPES buffer 6.0, and resazurin 0.001, agar 15.0, pH 7.5.	Thermal environment	Wery et al. [48]
Marine agar 2216	Peptone 5.0, yeast extract 1.0, ferric citrate 0.1, NaCl 19.45, MgCl_2_ 5.9, MgSO_4_ 3.24, CaCl_2_ 1.8, KCl 0.55, NaHCO_3_ 0.16, KBr 0.08, SrCl_2_ 0.034, H_3_BO_3_ 0.022, Na_2_SiO_3_ 0.004, NaF 0.0024, NH_4_NO_3_ 0.0016, Na_2_HPO_4_ 0.008, agar 15.0, pH 7.5.	Saline and alkaline environment	Zhang et al. [49]
MMB agar	Sodium acetate 0.5, yeast extract 0.5, Middlebrook 7H9 medium 4.7, casamino acids 0.5, sodium thiosulfate 0.5, NaCl 2, agar 15.0, pH 7.5.	Saline and alkaline environment	Jiang et al. [50]
Saline nutrient agar	Yeast extract 50, NaCl 81.0, MgSO_4_·7H_2_O 9.7, MgCl_2_·H_2_O 7.0, CaCl_2_ 3.6, KCl 2.0, NaHCO_3_ 0.06, NaBr 0.026, agar 15.0, pH 7.3.	Saline and alkaline environment	Rohban et al [51]
Modified ISP2 agar	Tryptone 10.0, yeast extract 5.0, glucose 5.0, agar 15.0, pH 2.5.	Acidic environment	Fang et al. [52]
Modified FeO agar	Component A: FeSO_4_·7H_2_O 7.0, distilled water 100 mL; component B: MgSO_4_·7H_2_O 0.7, (NH_4_)_2_SO_4_ 1.8, TSB medium 0.25, distilled water 650 mL; component C: agar 15.0, distilled water 250 mL, pH 2.5, the proportion of components A, B, and C is 2:13:5, pH 2.5.	Acidic environment	Johnson and Hallberg [53]
Modified 9K agar	Component A: (NH_4_)_2_SO_4_ 3, KCl 0.1, K_2_HPO_4_ 0.5, MgSO_4_·7H_2_O 0.5, Ca(NO_3_)_2_·H_2_O 0. 015, distilled water, 600 mL; component B: FeSO_4_·7H_2_O 22, distilled water 150 mL; component C: agar 15.0, distilled water 250 mL, the proportion of components A, B, and C is 12:3:5, pH 2.5.	Acidic environment	Zeinab et al. [54]
ISP agar	Component A: FeSO_4_·7H_2_O 7.0, distilled water 300 mL; component B: (NH_4_)_2_SO_4_ 6.0, KCl 0.2, MgSO_4_·7H_2_O 1.0, Ca(NO_3_)_2_ 0.02, distilled water 550 mL; component C: agar 15.0, distilled water 150 mL, the proportion of components A, B, and C is 6:11:3, pH 2.5.	Acidic environment	Manning [55]
PYGV agar	Peptone 0.25, yeast extract 0.25, nitrilotriacetic acid, 0.2, MgSO_4_·7H_2_O 0.6, CaCl_2_. 2H_2_O 0.07, Na_2_MoO_4_·2H_2_O 0.002, FeSO_4_·7H_2_O 0.002, agar 15.0, pH 7.5.	Cryogenic environment	Peeters et al. [56]
TSA agar	Casamino acids 17.0, papaic digest of soybean meal 3, dextrose 2.5, dipotassium hydrogen phosphate 2.5, NaCl 5.0, agar 15.0, pH 7.5.	Cryogenic environment	Ali et al. [57]
Trace salts	MgSO_4_·7H_2_O 3.0, MnSO_4_·2H_2_O 0.5, NaCl 1.0, FeSO_4_·7H_2_O 0.1, CoCl_2_ 0.1, CaCl_2_·2H_2_O 0.1, ZnCl_2_ 0.1, CuCl_2_·2H_2_O 0.02, AlK(SO_4_)_2_ 0.01, H₃BO₃ 0.01, Na_2_MoO_4_·2H_2_O 0.1, NiCl₂·6H₂O 0.03, Na_2_WO_4_·2H_2_O 0.03.	Thermal environment, xeric environment, saline and alkaline environment, cryogenic environment	Liu et al. [39]
Vitamin solution	Biotin 0.005, folic acid 0.005, pyridoxine hydrochloride 0.025, riboflavin 0.0125, thiamine 0.0125, nicotinic acid 0.0125, pantothenic acid 0.0125, cobalamin 0.005, *p*‐aminobenzoic acid 0.0125, thioctic acid 0.0125.	Thermal environment, xeric environment, saline and alkaline environment, cryogenic environment	Liu et al. [39]

^a^ The pH can be adjusted depending on the source of the samples.

#### Sample pretreatment

Samples from extreme environments can be approximately divided into three types, that is, solid (sediment, sand, ice, etc.), liquid (water and other liquid samples), and biofilms. For solid sample pretreatments, 2 g of sample are suspended into a conical flask containing 18 mL sterile water or 1/10 Reasoner's 2A (R2A) broth supplementary with glass beads, the suspension is incubated in an orbital shaker (200 rpm, 1 h). For liquid sample pretreatments, 2 mL of the sample is suspended into a conical flask containing 18 mL sterile water or 1/10 R2A broth. If the microbial biomass of the liquid sample is notably low, it is better to filter the sample using a 0.22‐μm filter membrane and then process the filtered biomass the same as for solid samples. Biofilm samples need to be ground fully first and then dealt with the same as for solid samples. *Note*: The 1/10 R2A broth can be used here instead of sterile normal saline because it contains pyruvate, amino acids, and so forth, which are known to be useful nutrients for resuscitating “viable but non‐culturable” strains [44, 58, 59]. However, if using 1/10 R2A broth instead of normal saline, there may be a risk of accelerating the growth of fast‐growing strains. In this case, reducing the shaking time will effectively inhibit the growth of microbes which respond rapidly to nutrient flushes.

#### Serial dilution

A 1 mL suspension is transferred from a conical flask to a 15 mL centrifuge tube with 9 mL sterile normal saline. Using pipettes, mix the liquid in the tube and then transfer 1 mL suspension to another 15 mL centrifuge tube with 9 mL sterile water; repeat these steps to dilute the samples to 10^−1^, 10^−2^, 10^−3^, and so forth. The diluted concentration suitable will change according to the biomass of each sample. Hence, it is better to carry out a pre‐experiment to confirm the appropriate diluted concentration before large‐scale formal experiments. *Note*: Cutting the sharp point of the pipette tips and shaking to mix suspensions will aid transfer.

#### Sample separation

A 100 μL suspension is transferred to the surface of solidified medium and spread evenly. During the whole process, the culture plates need to be kept 3–5 cm from the flame of a spirit lamp. Spread plates will be complete when no running droplets are observed after inclining the plates. Each plate needs to be recorded and marked with information including medium, sample type, temperature, dilution factor, and inoculation date on the cover of the Petri dish. The plates need to be placed face up for 2 h on a clean bench. After that, the plates should be turned upside‐down and placed in incubators. *Note*: Light is necessary for isolating some important taxa like *Cyanobacteria* [60] and it can also promote the isolation efficiency of some taxa, like, iron‐oxidizing bacteria [61]. These taxa are common groups that exist in extreme environments, therefore light also needs to be considered as an important parameter when incubate the isolation plates.

### Prokaryotic microorganism isolation and preservation

Materials and devices: Petri dish, spirit lamp, inoculation needles or bamboo sticks, glycerol and 2 mL cryogenic vials, inorganic salts, multivitamins, nystatin and DMSO, agar, precision balance, autoclave, refrigerator, ultralow temperature freezer, constant temperature incubator, and clean bench.

#### Colony picking

The isolation plates should be observed for growth after incubation for 3, 7, and 14 days (some samples such as permafrost soil may need longer times). After discrete colonies are clearly visible and evenly distributed on the surface of the medium, colony picking can start. Before picking, it is better to circle the target single clones on the bottom of the culture plate and mark a serial number. The target single colony is better to be well separated from other colonies to avoid being contaminated by other bacteria when picking. Colonies should be selected based on their color, size, transparency, edge features, and surface features as much as possible to avoid duplicate picks. The plate streaking method is used to transfer single colonies onto the isolation media for purification. After the isolated strains have established growth on the isolation media, they can be inoculated onto uniform culture media (generally Marine Agar 2216E for isolates from saline environments and R2A for other environments) to reduce workload. The biomass of the isolated strains on the isolation plates can be used for DNA extraction and preliminary identification, while those on the culture media plates can be used for preservation. *Note*: While very small colonies may look very similar, they are probably not fully mature, which means similar‐appearing colonies can be formed by different strains or species. Hence, it is appropriate to pick these very small colonies. Meanwhile, large colonies with similar appearance may not be the same strain and they differ in some metabolic functions so the duplicates removing steps need to be more careful. In addition, picking additional colonies from the isolation plates after different incubation times can recover more diverse isolates. At this time, the numbers on the bottom of plates will be helpful for recognizing the recently grown strains. We also find some archaea or other slow‐growing strains will form colonies very late [62, 63]. Hence, we recommend keep the isolation plates for 1 or 2 months after colony picking, and observed if there are slow‐growing strains form colonies.

#### Strain preservation

Sterile 2 mL cryogenic vials containing 1.5 mL glycerol (20%, v/v) are used for the preservation of isolated pure cultures. Using sterile inoculation needles pick a suitable amount of fresh biomass (approximately mung bean size) and spread strains evenly on the tube wall. Incline the tube so that the glycerol covers the spreading area and lightly shake to form a suspension. The suspension should first be prefrozen at −20°C for 2 h and then preserved at −80°C. *Note*: Spreading and scraping avoids more dramatic actions which may lead to extraneous contamination. Prokaryotes from liquid culture can also be used for strain preservation after forming suspension within glycerol (final concentration: 20%, v/v).

### Preliminary strain identification

Materials and devices: pipette tips (20 μL, 200 μL, 1 mL), polymerase chain reaction (PCR) tube, scissors, Chelex‐100, Tris‐EDTA buffer, PCR master mix, and PCR gene amplification instrument.

#### DNA extraction

For large‐scale DNA extraction experiments, 10% Chelex suspension is recommended (10 g Chelex‐100 add to 90 mL Tris‐EDTA buffer). The steps are as follows: (1) Shake the Chelex suspension, then draw up 50 μL suspension using a cutoff pipette tip and transfer to the PCR tube. (2) Add a small amount of biomass to the PCR tube and mixed well with the Chelex. (3) Place the PCR tube in the PCR instrument or water bath kettle and heat at 99°C for 30 min. (4) Briefly centrifuge to remove the cell debris and let the Chelex settle to the bottom of the PCR tube; the supernatant can be used for the PCR amplification experiment. It may be hard to extract DNA from certain strains containing excessive pigment or extracellular polymeric substances using Chelex suspension. Then we recommend using the phenol‐chloroform method to extract DNA and the specific steps are described in Li et al. [64].

#### Amplification, sequencing, and identification

The 16S ribosomal RNA (rRNA) gene is used for preliminary microbial identification. The primers used for 16S rRNA gene amplification are 27F (5′‐CAGAGTTTGATCCTGGCT‐3′) and 1492R (5′‐AGGAGGTGATCCAGCCGCA‐3′) for Bacteria, or P1 (5′‐ATTCCGGTTGATCCTGCCGGA‐3′) and P2 (5′‐AGGAGGTGATCCAGCCGCAG‐3′) for archaea [65]. The PCR is performed according to the following program: 94°C initial denaturation for 5 min, followed by 32 cycles of denaturation at 94°C for 45 s, annealing at 56°C for 30 s, and extension at 72°C for 1 min 30 s, and a final extension at 72°C for 10 min. The PCR products can then be sent for sequencing using Sanger sequencing technology. The obtained sequences can be identified using the EzBioCloud server databases [66] or NCBI Basic Local Alignment Search Tool [67]. Notably, EzBioCloud's 16S‐based ID service (https://www.ezbiocloud.net/identify) provides reliable similarity‐based searches against quality‐controlled databases of 16S rRNA gene sequences but need to be identified one by one. To reduce the heavy workload of submitting individual sequences, we developed a bioinformatic tool for easily analyzing 16S rRNA gene sequences against the EzBioCloud database that can automatically submit, identify, and dissect sequences (https://github.com/lianzhh/easy_EzBioCloud). The top‐hit information for each identification will be saved and a pie chart generated for analyzing the cultivable microbial diversity when the identification is complete. Generally, if the similarity of 16S rRNA gene sequence between an isolate and the top‐hit type strain with a validly published name is lower than 98.65%, we will consider this isolate as a putative novel species [68]. *Note*: Because of the characteristics of Sanger sequencing technology, the beginning and ends of the sequences obtained by this technology are unreliable. The quality of Sanger sequencing reads should be visualized and checked by tools like Molecular Evolutionary Genetics Analysis software (https://www.megasoftware.net/) or BioEdit software to decide the location of primer sequences to be cut before identification.

## SPECIFIC STRATEGIES FOR DIFFERENT EXTREMOPHILES ISOLATION

**Figure 2:**
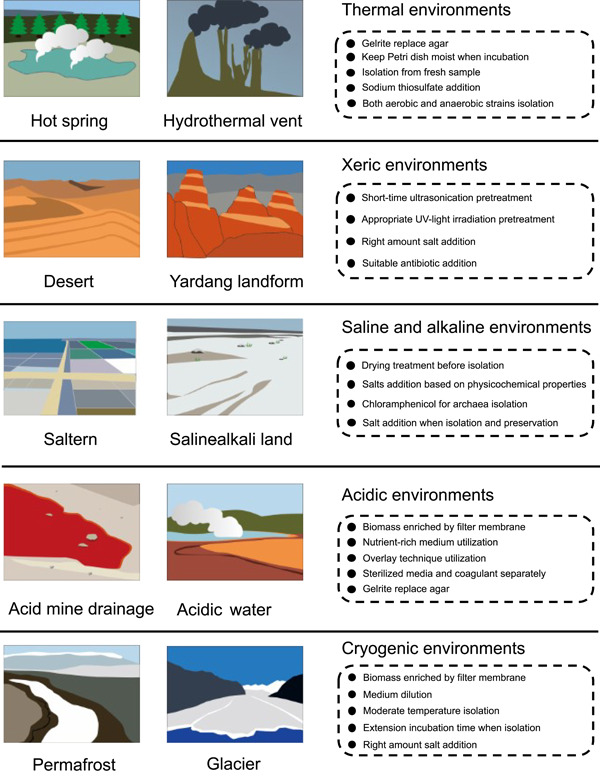
Strategies and tips for isolation of microbes from different kinds of extreme environments.

Extreme environments have unique microbial communities due to their distinctive limiting factors. Hence, diverse isolation strategies and matters need attention when exploring different environments (Figure 2).

### Thermal environments

Thermal environments mainly include terrestrial hot springs and deep‐sea hydrothermal vents. High temperature is one of the major factors restricting life in these places. To isolate microorganisms successfully, enhanced isolation temperatures are necessary. However, high temperatures will denature and liquefy agar, so if the isolation and culture temperature is higher than 60°C, 0.8% gelrite should be used instead of agar. High temperatures will also make the media dry out, so isolation plates which require long incubation times need to be packed into plastic bags with a Petri dish full of water to keep the incubation moist. Moreover, the microbial isolation process of the former study suggested that the biodiversity of hot spring samples will decline rapidly after cryogenic preservation [47]. Therefore, the sample isolation process should be carried out while the sample is fresh; if the sample needs to be stored, normal temperature is better than low temperature. As a dominant group in hot springs, the members of the genus *Thermus* have attracted extensive attention: according to a previous study, the addition of sodium thiosulfate to the media will promote efficiency in isolation of this group [69]. The nutrient requirements of microorganisms in and around hydrothermal vents are very complex [70]. Hence, a single type of medium will often not perform well, so that the media selected to isolate prokaryotic microorganisms from such sources should be considered to provide various nutritional requirements [48]. In addition, thermophilic environments often support both aerobic and anaerobic strains at the same time and therefore oxygenation also needs to be considered when isolating strains.

### Xeric environments

Desert and Yardang landforms are representative xeric environments which, due to drought and high ultraviolet solar radiation, have been regarded as sterile lands. However, even the Atacama Desert, which has extreme natural conditions, still has a huge number of diverse microbes [71]. Compared with other extreme environments, the sample pretreatment strategies for desert samples need to be more drastic. Generally, ultrasonication at 45 kHz for 2 min is a reliable sample pretreatment strategy that can effectively restrain the growth of faster‐growing strains. Alternatively, we treated desert samples using UV‐light exposure on a clean bench for 10 min and acquired higher pure culture diversity compared with normal cultivation methods which had similar effects to previous study [72]. Moreover, although the salt concentration is not typically high in desert environments, some strains recovered can tolerate extreme salt conditions. Indeed, we have isolated and described a species, *Saccharopolyspora deserti*, which can tolerate up to 22% NaCl (w/v; ≈3.8 M) [73]. Thus, adding varying levels of salt to the isolation media may help isolate more unique strains. Desert samples contain a very high proportion and diversity of members of the phylum *Actinomycetota*, especially *Streptomyces*, suggesting that desert habitats might be treasure troves for bioprospecting for secondary metabolites. Nalidixic acid (25 mg/L), cycloheximide (25 mg/L), and potassium dichromate (25 mg/L) can be added to the isolation media to inhibit the growth of competitor bacteria and so significantly promote the isolation of members of *Actinomycetota* [37].

### Saline and alkaline environments

Halophiles and alkaliphiles live in hypersaline environments, such as salt lake, marine saltern, and saline‐alkali land. Typically, saline and alkaline samples do not need air drying before isolation, but air drying during isolation is a useful method to recover higher proportions of halophilic or alkaliphilic *Actinomycetota* and archaea [74]. It is also noted that different saline environments have distinct saline compositions, so other salts such as KCl or MgCl_2_ may need to be added to the isolation media according to the physicochemical properties of the samples. In addition, high concentrations of salt may form precipitates with organic components in media when sterilized and therefore salt stocks and media should be sterilized separately when this phenomenon happens. Among the microbial communities of saline environments, halophilic archaea belonging to the *Euryarchaeota* are noted to form a significant proportion of the microbiota of halophilic ecosystems [75]. Adding chloramphenicol (25 mg/L) can effectively inhibit the growth of halophilic bacteria such as *Halomonas* spp. to promote efficiency in isolating halophilic archaea. In addition, microorganisms living in these environments often require a particular salt concentration to maintain the osmotic potential that is required for their normal physiology. Therefore, as well as in isolation and cultivation media, NaCl (5%–10%) should be added to the solutions used for sample pretreatment and glycerol used for preservation to sustain these strains.

### Acidic environments

AMD is a typical acidic environment which not only has a very low pH but also always contains a very high concentration of heavy metal ions. Hence, microbial species richness in AMD environments is restricted by these stressful conditions, which means that at the sample pretreatment step, liquid samples of AMD need to be enriched using a 0.22‐μm filter membrane and the sediment samples need reduced dilution factors. Generally, media used for isolating strains from AMD samples contain little or no organic nutrients. However, we tested a modified ISP2 medium (Table 1), a nutrient‐rich medium, to isolate strains from AMD sediment samples with good results, acquiring many kinds of Actinomycetota [52]. Also, an approach called the “overlay technique” has been used to facilitate the growth of a broad range of known moderately thermophilic and mesophilic acidophiles [53]. Briefly, this approach is performed by first pouring medium with a selected heterotrophic acidophile as thin gels in sterile Petri plates and adding a second layer of the sterile medium after the bottom medium is solidified. With this, toxic organic materials, invariably present in agar‐based gelling agents, and also produced during plate incubation, are removed by the heterotrophic acidophile which is incorporated into the lower layer of a two‐layered gel. The specific steps of this approach can be found in Johnson and Hallberg [53]. In addition, it is worth noting that, as for high temperatures, low pH may denature agar which makes it hard to solidify. Therefore, media and agar for isolating acidophiles are necessary sterilized separately and agar should be replaced with gelrite if necessary.

### Cryogenic environments

Glacier and permafrost are the typical cryogenic environments, which maintain temperatures below 0°C all the year round. Glaciers, which include continental ice caps and mountain glaciers, cover about 10% of the Earth's surface [76]. Both of these environments are underpopulated with microbial biomass, and so it is better to melt ice samples into water and enrich the microorganisms using a 0.22 μm filter membrane during the sample pretreatment step. Similar to glaciers, permafrost is also divided into high‐altitude permafrost and high‐latitude permafrost. Their physicochemical properties and nutritional conditions, and also their microbial community composition, are different, which means isolation plans need to consider these differences. For example, in China, permafrost in Qinghai–Tibet Plateau contains a lower concentration of organic matter and inorganic salt or microbial biomass compared with permafrost in the northeast of China [77]. Hence, the media used for isolating strains from Qinghai–Tibet Plateau permafrost samples recommends to be diluted in 1/4 R2A or 1/10 TSA. Most cultured microorganisms from cryogenic environments appear to be primarily psychrotolerant rather than psychrophilic, and the optimum growth temperature of these strains is from 12°C to 25°C [78, 79, 80]. Hence, at the time of isolation, in addition to low temperatures, such as 0°C or 4°C, moderate temperatures, such as 16°C or 25°C should also be chosen. For the same reason, permafrost samples can be kept at 4°C for several weeks to increase the number of culturable bacteria [81]. We have found some psychrophiles grow very slowly and needed several months to form colonies at a low temperature, so the incubation time of the isolation plates needs to be lengthened to at least 2 months at lower temperatures, such as 4°C. Similar to strains from desert environments, many psychrophiles isolated from glaciers or permafrost have been found to tolerate a range of salt concentrations [77, 80]. Hence, adding 3%–5% (w/v) NaCl to the isolation media may improve strain recovery.

### Anaerobic extremophiles

Anaerobic extremophiles are widely distributed and play an important role in extreme environments [3, 17, 82]. However, compared with aerobic microbes, there are more challenges to isolate anaerobes because the isolation must be performed in anoxic conditions. Therefore, the key point for isolating these strains is to remove the oxygen in the medium and keep the anaerobic environment. Hungate roll‐tube technique is a traditional method for isolating anaerobes, and it is suitable to culture strict anaerobes, such as methanogenic bacteria [83]. Alternatively, the anaerobic chamber technique has the advantage that Petri dishes can be used, which make replica plating feasible and successful in cultivating many anaerobes [39]. In brief, for the anaerobic chamber technique, the medium was prepared under aerobic conditions, and immediately transferred to the anaerobic workstation containing a gas phase of N_2_:H_2_:CO_2_ (8:1:1) after sterilization. Then, the medium was poured into the Petri dishes together with a sterile reducing agent under anaerobic condition [84]. Finally, samples were homogenized and serially diluted in sterile water solution to 10^−3^, aliquots (100 μL) were then spread plated onto the isolation plates and incubated anaerobically. It is worth emphasizing that samples should be enriched to obtain the special anaerobic community. Serum bottles with a butyl rubber cap that can reduce the influence of oxygen [85], which also can be used to enlarge the biomass of microorganisms after achieving a pure culture. Furthermore, aiming for the slow‐growing of anaerobic strains, microfluidic systems or other single‐cell cultivation methods would be useful for dividing strains into single cells, and then the slow‐growing strains will not be influenced by fast‐growing strains [86, 87].

## MULTI‐OMICS‐GUIDED MICROBIAL CULTIVATION

**Figure 3:**
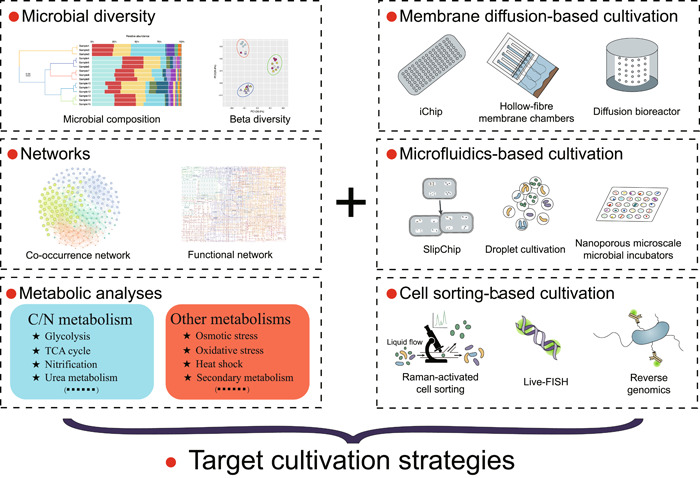
Schematic of targeted microbial cultivation strategies. Information from multi‐omics approaches can be combined with other techniques and innovative cultivation methods to provide the framework necessary for rationally designing targeted cultivation strategies. iChip, isolation chip; TCA, tricarboxylic acid.

**Figure 4:**
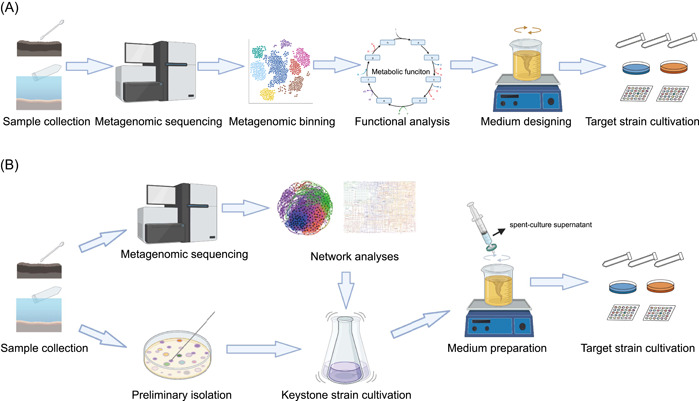
Two examples for multi‐omics‐guided microbial cultivation approaches. (A) Function guiding microorganisms cultivation and (B) network guiding microorganisms cultivation.

The cultivation strategies we introduced above are untargeted and used for all prokaryotic microorganisms living in extreme environments. With the development of diverse high‐throughput sequencing technologies and algorithms, some uncultured microorganisms known to possess particular functions or notable positions in phylogenetic analyses have been found through analysis of metagenome‐assembled genomes (MAGs), single‐amplified genomes (SAGs) or other omics methods. Moreover, taxa described from MAGs and SAGs can now be named using the SeqCode [88]. Despite advances in the variety of high‐throughput sequencing techniques, there is always a need for microbial cultivation to provide material for the study of living microorganisms (e.g., physiology). This is inherently a challenging task [21]. Fortunately, information from SAGs, MAGs, and other approaches can be combined with that from other techniques (microautoradiography, fluorescence in situ hybridization, nanometer‐scale secondary ion mass spectrometry, Raman, etc.) and innovative cultivation methods (membrane diffusion [89]/cell sorting [90]/microfluidics‐based cultivation methods [91]) to provide the framework necessary for rationally designing targeted cultivation strategies (Figure 3). For instance, Chen et al. used 39 SAGs/MAGs of Micrarchaeota and Parvarchaeota to infer their metabolic potential, and then successfully designed a low‐oxygen cultivation strategy to confirm their microaerobic/anaerobic lifestyle [92]. In addition, innovative cultivation methods (Figure 3), such as isolation chip technology that allows microorganisms to exchange metabolites with the environment and other species [89], can increase the number of cultivable microorganisms. Besides, an immunomagnetic bead‐enriched culturomics method is reported and has the ability to specifically isolate potential pathobionts for a particular disease of interest [93]. It is worth noting that microbial interactions have traditionally been ignored in cultivation method design, which might be one key reason that many as‐yet‐uncultivated microbes remain uncultivable. Therefore, data‐supported networks, such as co‐occurrence networks from 16S rRNA gene data sets [94], and functional networks from multi‐omics data, should provide great opportunities for guiding the design of cultivation strategies. Here, we share our two targeted microbial isolation approaches where multi‐omics is used to guide the targeted cultivation strategies (Figure 4).

The phylum *Actinomycetota* is one of the largest taxonomic units in the domain Bacteria, members of which are well known for their extensive secondary metabolisms [95]. Until now, most knowledge of this group has been based on pure cultures and the ecological functions of uncultivated *Actinomycetota* remain mysterious. Through analyzing metagenomic data, we found 35 high‐quality MAGs which represented three novel actinobacterial classes (*Ca*. Geothermincolia, *Ca*. Humimicrobiia, and *Ca*. Aquicultoria) [96]. Genomic inference of these MAGs showed that they contain the genes coding for hydrogenases, energy‐conserving mechanisms, acetogens, and Wood–Ljungdahl pathway, indicating they are putative homoacetogens. According to the genomic investigation, anaerobic enrichments with different treatments (G55H: the ratio of N_2_:H_2_:CO_2_ was 5:4:1; (2) G55HB: the ratio of N_2_:H_2_:CO_2_ was 5:4:1 with 20 mM 2‐bromoethanesulfonate) were performed to enrich this group [96]. During the process of enrichment, the concentrations of H_2_, CO_2_, and acetate were tested to verify if homoacetogenesis was taking place. Amplicon sequencing was used for understanding changes in the microbial community structure. After 120‐days enrichment, we found the proportion of the target *Actinomycetota* had increased drastically, indicating the success of our multi‐omics‐guided enrichment strategy for *Ca*. Geothermincolia. In general, omics data‐guided strain enrichment works well when applied to potential functional microbial groups (Figure 4A). However, it still faces difficulties in yielding pure cultures, and novel cultivation methods are needed, such as cell sorting‐based cultivation, microfluidics‐based cultivation, and membrane diffusion‐based cultivation [28].

Another case relates to network‐directed extremophile isolation (Figure 4B) [96]. Through high‐throughput sequencing and cooccurrence network analysis of Tibet and Yunnan hot spring samples, we found less abundant operational taxonomic units (OTUs) belonging to the genus *Tepidimonas* (relative abundant, 0.014%) had a high‐degree centricity (key nodes), while dominant OTUs from the genus *Chloroflexus* (relative abundant, 13.9%) formed the peripheral vertexes. On the basis of this result, we hypothesized that the microbial community was sustained by the rare members with high centrality by facilitating the basic metabolisms of abundant members. Before testing our hypothesis, we confirmed that these two members have the potential for metabolic exchange according to the function network analysis from genomic data. Therefore, we used R2A containing 10% spent‐culture supernatant from culture of a previously isolated *Tepidimonas* sp. to target isolation of the dominant *Chloroflexota* [97]. After isolation and sequencing, the 16S rRNA gene fingerprinting characterized the majority of the colonies isolated from these samples as previously uncultivated *Chloroflexota*, which included representatives of 36 potential novel species and one novel class. These results indicate that using high‐throughput sequencing and co‐occurrence/function networks for finding keystone species and isolating microorganisms according to their interaction relationship may be an effective method in extremophiles cultivation.

## CONCLUSION

Compared with culture‐independent approaches, microbial isolation and cultivation are undoubtedly time‐consuming and tedious, but microbial cultures are undoubtedly useful for deciphering microbial physiology, testing multi‐omics‐based functional hypotheses, and paving the way towards applications in biotechnology, and so forth. Microorganisms from extreme environments are more difficult to isolate, compared with those from less challenging environments, due to their specific adaptions and metabolisms. Here, we provide a straightforward protocol for efficiently isolating prokaryotes from different extreme habitats and propose the use of multi‐omics‐guided microbial cultivation approaches for culturing these as‐yet‐uncultivated microorganisms, which makes isolation work for samples from these environments easier and more efficient, thereby increasing the value of these microbial resources.

## AUTHOR CONTRIBUTIONS

Zi‐Wen Yang and Jian‐Yu Jiao wrote the draft manuscript and revised the figures. Zheng‐Han Lian developed a bioinformatic tool. Lan Liu and Bao‐Zhu Fang revised the manuscript. Wen‐Jun Li and Jian‐Yu Jiao jointly conceived this study. All authors have read and approved the final manuscript.

## CONFLICT OF INTEREST STATEMENT

The authors declare no conflicts of interest.

## Data Availability

The bioinformatic tool for easily analyzing 16S rRNA gene sequences against the EzBioCloud database is available at https://github.com/lianzhh/easy_EzBioCloud. Supplementary materials (figures, tables, scripts, graphical abstracts, slides, videos, Chinese translated versions, and updated materials) may be found in the online DOI or iMeta Science https://www.imeta.science/.
